# Equine Alphaherpesviruses Require Activation of the Small GTPases Rac1 and Cdc42 for Intracellular Transport

**DOI:** 10.3390/microorganisms8071013

**Published:** 2020-07-07

**Authors:** Oleksandr Kolyvushko, Maximilian A. Kelch, Nikolaus Osterrieder, Walid Azab

**Affiliations:** Institut für Virologie, Robert von Ostertag-Haus, Zentrum für Infektionsmedizin, Freie Universität Berlin, Robert-von-Ostertag-Str. 7-13, 14163 Berlin, Germany; olek@zedat.fu-berlin.de (O.K.); maximilian-alexander.kelch@charite.de (M.A.K.); no.34@fu-berlin.de (N.O.)

**Keywords:** alphaherpesvirus, EHV, small GTPase, Cdc42, Rac1, cell entry, host pathogen interaction, intracellular transport

## Abstract

Viruses utilize host cell signaling to facilitate productive infection. Equine herpesvirus type 1 (EHV-1) has been shown to activate Ca2+ release and phospholipase C upon contact with α4β1 integrins on the cell surface. Signaling molecules, including small GTPases, have been shown to be activated downstream of Ca2+ release, and modulate virus entry, membrane remodeling and intracellular transport. In this study, we show that EHV-1 activates the small GTPases Rac1 and Cdc42 during infection. The activation of Rac1 and Cdc42 is necessary for virus-induced acetylation of tubulin, effective viral transport to the nucleus, and cell-to-cell spread. We also show that inhibitors of Rac1 and Cdc42 did not block virus entry, but inhibited overall virus infection. The Rac1 and Cdc42 signaling is presumably orthogonal to Ca2+ release, since Rac1 and Cdc42 inhibitors affected the infection of both EHV-1 and EHV-4, which do not bind to integrins.

## 1. Introduction

Cellular functions are governed by complex signaling networks, and regulation is achieved by countless molecules, most of which are proteins [1]. Small GTPases are cellular signaling proteins that hydrolyze GTP and transduce the signal by changing the dynamics of interaction with other cellular proteins in the phosphorylated (GTP) or dephosphorylated (GDP) bound states. The regulation of small GTPases is achieved by several factors, the most important being guanine nucleotide exchange factor (GEFs), GTPase-activating proteins (GAPs), and guanine nucleotide dissociation inhibitors (GDIs) [2]. The role of GEFs is to promote release of GDP and allow the binding of GTP. GDIs play an opposite role, by preventing GDP release from the small GTPase molecule. GAPs are responsible for activation of GTP hydrolysis [3,4]. Additionally, other factors, such as cytoplasmic Ca2+, may facilitate the activation of small GTPases, as is the case in lamellipodia formation by platelets [5]. Small GTPases are understood to be in an active state when GTP is bound, and inactive when GDP is bound [4]. The Rho family of small GTPases was first discovered through transcriptomic screens, as they were overexpressed in cancer cells, and later described to play a role in a number of important cellular processes, such as morphological changes, reorganization of the cytoskeleton, cell cycle regulation, growth, motility, and adhesion [3,6]. The dysregulation of small GTPases may lead to disease and cancerous transformation, abnormal patterns of expression or mutations of GEFs; GDIs and GAPs are associated with different cancer types [2,7,8,9]. Members of Rho family small GTPases, such as Rac1 and Cdc42, were described to play roles in virus infection [10,11,12].

Viruses have evolved mechanisms to hijack cellular signaling, in order to facilitate infection. The *Alphaherpesvirinae* subfamily includes many pathogens that are of great importance to animal and human health, including equine herpesviruses 1 and 4 (EHV-1 and EHV-4). Cell entry of the two closely related alphaherpesviruses EHV-1 and EHV-4 exhibits differences, even though MHC class I molecules are the entry receptor for both [13,14]. Following receptor binding that is mediated by glycoprotein D (gD), EHV-1 gH binds to α4β1-integrin and induces cellular signaling cascades, resulting in virus fusion with the plasma membrane. The disruption of gH-α4β1-integrin interaction results in the inhibition of signaling cascades and re-routing of the virus to a caveolin/raft-dependent endocytic pathway. On the other hand, EHV-4 cannot interact with cell surface integrins, and enters equine cells through the caveolin/raft-dependent endocytic pathway. In particular, EHV-1 is able to induce signal transduction inside the infected cell that leads to the activation of phospholipase C, the release of inositol triphosphate, Ca2+ release from endoplasmic reticulum, after interaction with α4β1-integrins on the surface of the cells. This signaling cascade is necessary for fusion at the plasma membrane [15]; however, the exact mechanism that facilitates virus entry is still unknown. The investigation of cellular signaling may lead to better understanding of host-pathogen interaction. Small GTPases were described to be activated downstream of Ca2+ release, and are involved in cellular processes such as cytoskeleton remodeling, membrane fusion and intracellular transport. These properties make small GTPases a good candidate to further investigate the signaling cascade induced by EHV-1 [16,17,18].

In the current study, we tested the hypothesis that small GTPases play a role in EHV-1 infection, with assays based on chemical inhibitors of small GTPases, cell-to-cell spread, and FRET biosensor GTPase activation assays. We further identified specific steps of the infection process, at which Rac1 and Cdc42 play a crucial role. We identified that Rac1 and Cdc42 small GTPases activation is required for the intracellular transport of EHV-1 through the acetylation of microtubules.

## 2. Materials and Methods

### 2.1. Cells and Viruses

Equine dermal (ED) cells (CCLV-RIE 1222, Federal Research Institute for Animal Health, Germany) were cultivated in Iscove’s modified Dulbecco’s medium (IMDM) (Invitrogen, Carlsbad, USA), supplemented with 20% fetal bovine serum (FBS; Pan - Biotech GmbH, Aidenbach, Germany), 100 U/mL penicillin (Roth, Karlsruhe, Germany), 100 μg/mL streptomycin (Alfa Aesar, Haverhill, USA), 1 mM sodium pyruvate (Pan - Biotech GmbH) and 1x nonessential amino acids (Pan - Biotech GmbH). Human embryonic kidney (293T) cells were cultured in Dulbecco’s modified Eagle’s medium (DMEM) (Biochrom, Cambridge, UK), and supplemented with 10% FBS (Pan - Biotech GmbH), 100 U/mL penicillin (Roth), and 100 μg/mL streptomycin (Alfa Aesar). Cells were grown at a temperature of 37 °C and a 5% CO_2_ atmosphere.

EHV-1 strain RacL11 (L11-RFP), expressing red fluorescent protein (RFP), fused to the small capsid protein VP26 [15], EHV-1 gH4 [19]—EHV-1 expressing gH from EHV-4 that cannot bind to α4β1 integrins (L11-gH4), EHV-1 gHS440A [19] that harbors 3 amino acid substitutions in the gH-integrin binding motif that renders the virus unable to bind to α4β1 integrins, and the EHV-4 strain TH20p [20] was used in this study. All viruses express the enhanced green fluorescent protein (eGFP) for the rapid identification of infected cells. Viruses were reconstituted by the transfection of 2 μg of bacterial artificial chromosome (BAC) DNA into 293T cells using polyethylenimine (PEI; 408727, Sigma-Aldrich, St. Louis, MI, USA). Viruses harvested form 293T cells were then passaged on ED cells. For all experiments, only viruses grown on ED cells were used. For UV-inactivation, 150 μL of virus containing media was placed in a 5-cm cell culture dish and exposed to 30 ss at a power setting of 600, using a UV DNA crosslinker at 254 nm and 8 Watt UV tubes (Analytik Jena, Jena, Germany) [21]. Such parameters were sufficient to efficiently inactivate an infectious virus, as determined by back titration.

### 2.2. Inhibitors

RhoA Inhibitor I based on a purified C3 Transferase (dissolved in water; Cat. # CT04, Cytoskeleton, Inc.), and a Rho/Rac/Cdc42 Activator I (dissolved in water; Cat. # CN04, Cytoskeleton, Inc.) were used at final concentrations of 2 μg/mL, following the manufacturer’s instructions. Rac1-specific inhibitor NSC 23766 (dissolved in water; ab142161, Abcam, Cambridge, UK) and Cdc42 specific inhibitor ML-141 (dissolved in DMSO; ab145603, Abcam) [22] were used at final concentrations of 100 μM and 80 μM, respectively. Stocks of ML-141 were dissolved in DMSO and the final concentration of DMSO in the media was 0.4% [23]. Target cells were serum-starved for 1 h and incubated for 3 h in the presence of inhibitors before further treatment, unless otherwise specified.

### 2.3. Cytotoxicity Assay

The cytotoxic effects of inhibitors at selected concentrations on ED cells were assessed using the WTS-1 assay (Cayman Chemicals No. 10008883) after 24 and 48 h, as described before [24,25]. In short, cells seeded in a 96-well plate were cultivated for 24 or 48 h at 37 °C under a 5% CO2 atmosphere in media, with the designated concentration of inhibitors. Negative control consisted of cells without the addition of inhibitors. Cells treated for 30 seconds with a 30% solution of hydrogen peroxide (Sigma No. H1009) were used as a positive control. Cells were treated for 24 or 48 h with the inhibitors as specified above, before the WTS-1 reagent was added to the media of each well and incubation for 2 more hours. Absorbance at 450 nm was measured for each sample using a microplate reader (BioTek Instruments, Winooski, VT, USA).

### 2.4. Flow Cytometry

ED cells were seeded in a 96-well plate, such that, on the day of the experiment, 2.5 × 10^4^ cells were present in each well. On the day of the experiment, media were replaced with media containing one of the chemical inhibitors, the Rho/Rac/Cdc42 Activator I, or regular medium as a negative control. Cells were incubated for 3 h and then placed on ice for 10 min. EHV-1, EHV-1 gH4, or EHV-1 gHS440A were added at a multiplicity of infection (MOI) of 1. Cells were kept with the virus on ice for 1 h, to synchronize the infection, then cells were shifted to 37 °C. After 1 h, cells were treated with ice-cold citrate buffer (pH 3) for 1.5 min, to neutralize unpenetrated viruses [25], followed by 2 h incubation with fresh media that also contained the inhibitors or the activator. At this point (in total: 3 h after the temperature shift) 10,000 cells were analyzed for eGFP expression by flow cytometry (CytoFLEX, Beckman Coulter, Pasadena, USA). Notably, eGFP expression in cells without inhibitor treatment was considered the baseline of infection and set at 100%. Flow cytometry data was analyzed using the FlowJo software (FlowJo, LLC, Ashland, USA) to ensure gating consistency. Each treatment had 3 technical replicates per plate, and the complete experiment was repeated three independent times.

### 2.5. Plaque Assay

ED cells were grown in a 6-well plate (1 × 10^5^ cells per well). ED cells were treated with one of the chemical inhibitors, or mock-treated for 3 h. Cells were then placed on ice for 10 min and EHV-1, EHV-1 gH4, EHV-1 gHS440A, or EHV-4 were added at a titer of 100 PFU (plaque forming unit) per well. Attachment was allowed for 1 h to synchronize the infection. To promote virus penetration, cells were incubated at 37 °C for 60 min before they were subjected to citrate treatment (see above) and overlaid with DMEM containing 0.5% methylcellulose in the presence or absence of the inhibitors. Plaques were counted and diameter was measured in a blinded fashion after 48 h of infection at 37 °C using inverted fluorescent microscope (Zeiss Axiovert 100, Zeiss, Oberkochen, Germany), provided with a monochromatic camera (ZEISS Axiocam 305 mono). All taken images were analyzed with Fiji image analysis software. Fifty or all available (if less than 50) plaques were measured for each of the three independent replicates. If no plaques (but only single infected cells) were found per run, plaque diameter was considered to be equal to zero. Single infected cells were not considered plaques. The average of the plaque diameter of each of the three repeats was used for statistical analysis.

### 2.6. Virus Localization and Immunofluorescence

To quantify and visualize virus localization, 1 × 10^4^ ED cells were seeded on a coverslip, in a 24-well plate. Cells were treated with each individual inhibitor, or mock-treated for 3 h. Before addition of EHV-1, cells were placed on ice for 10 min and EHV-1 was added at a MOI of 1. Virus attachment was allowed to proceed for 1 h on ice, followed by a temperature shift to 37 °C and incubation for 30 min (virus-membrane localization setup), 3 h (virus-endosome localization setup), or 6 h (virus-nucleus localization setup). After the given incubation time, infected cells were fixed with 4% paraformaldehyde (PFA) and stained in one of the three following ways. (i) To visualize the external leaflet of the plasma membrane, cells fixed at 30 min after infection were stained with lectin-FITC conjugate (16441, Sigma-Aldrich). (ii) To examine virus colocalization with endosomes, ED cells were fixed at 3 h post infection (HPI), permeabilized with permeabilization buffer (2% BSA, 0.2% Triton-X 100 in PBS) for 20 min, and stained for 1 h with polyclonal antibodies against LAMP1, EEA1, or caveolin-1 (ab24170, ab2900, ab2910, Abcam, respectively) diluted 1:200 in antibody-dilution buffer (2% BSA, 0.1% Tween-20 in PBS). Secondary goat anti-rabbit IgG Alexa Fluor 647 (Abcam ab150079) was diluted 1:2000 in antibody-dilution buffer and added to the cells. (iii) DAPI was used to visualize the cell nucleus. The cells were examined with a confocal laser scanning Nikon Eclipse Ti Visitron microscope (Visitron Systems GmbH, Puchheim, Germany). Images were analyzed with VisiVIEW imaging software (Visitron Systems GmbH). Virus particles were counted using ImageJ (NIH), according to the following strategy: (i) for the virus-membrane localization setup, all virus particles on (or very close to) the cell membrane were considered to be localizing with the cell membrane; (ii) for the virus-endosome localization setup, all virus particles were considered to be colocalizing with the endosome or being spatially separated from the endosomal markers; (iii) for the virus-nuclear localization setup, all virus particles localized within the DAPI-stained nucleus were considered to exhibit nuclear localization. All other virus particles that were present in the cells and did not fall within one of the 3-setups were considered to localize to the cytoplasm. One-hundred virus particles were counted and categorized manually in a blinded fashion, for each of the 3 independent replicates of the experiments.

### 2.7. Ratiometric Fluorescence Resonance Energy Transfer (FRET)

ED cells were grown in an 8-Well µ-Slide (Ibidi) and transfected with pRaichu-Rac1 or pRaichu-Cdc42 plasmids [26,27], using a Xfect Single Shots transfection reagent (Clontech, Mountain View, CA, USA). At 24-h after transfection, wells were analyzed with an inverted fluorescent microscope (Zeiss) at 488 nm, to find which wells had been successfully transfected with the biosensors. Media was replaced with live cell imaging media (Gibco 21063029, Life Technologies, Carlsbad, CA, USA) without serum. Cells were placed on ice for 2 h, and then into a climate control chamber (37 °C, 80–90% humidity, and 5% CO2) of the Leica SP8 CLSM. Cells were allowed to equilibrate to the temperature and CO_2_ concentration of the chamber for 30 min. Cells were illuminated with Argon laser combined with AOBS beam splitter and the 63x/1.4 HC PL APO CS2 oil immersion objective was used. The excitation beam was tuned to a wavelength of 433 nm; the reporter wavelength was 457 for the donor (CFP) and 527 for the acceptor (FRET) [26,27]. UV-inactivated EHV-1 was added to cells during imaging at a MOI of 10, within the second minute of imaging. Both reporter and acceptor emissions were recorded simultaneously with two gallium arsenide phosphide photomultiplier (PMT) sensors. Cells were imaged with a frequency of 2 Hz, to reduce phototoxicity. Average intensity for the region of interest (Raichu biosensor expressing ED cell) was measured for each channel and time point, and the ratio of FRET/CFP was calculated and plotted as a moving average of 20 points. The experiment was repeated 3 independent times for each biosensor.

### 2.8. Immunoblotting

Immunoblotting was performed to quantify the proportion of acetylated tubulin [28]. On the day of the experiment, 4 × 10^5^ 293T cells in a 12-well plate were serum-starved for 1 h. Cells were treated with small GTPase inhibitors in DMEM for 3 h in a cell culture incubator. The inhibitor-treated 293T cells were infected with EHV-1 at a MOI of 10, for 1 h and lysed on ice as described before [29]. Paclitaxel (T7191 Sigma-Aldrich), a chemical inducer of microtubule acetylation, was used as a positive control. Briefly, 293T cells were serum-starved for 1 h, overlaid with serum-free DMEM containing Paclitaxel at a final concentration of 10 μM for 1 h, and finally, lysed on ice. Cell lysis was achieved by heating the sample to 98 °C in sodium dodecyl sulfate (SDS) sample loading buffer (1 M Tris-HCl (pH 6.8), 0.8% sodium dodecyl sulfate (SDS), 40% glycerol, 0.2% β-mercaptoethanol, 0.02% bromophenol blue) for 10 min. A 12% SDS-polyacrylamide gel was used to separate the proteins based on their molecular weight. Semi-dry transfer was used to transfer proteins to the polyvinylidene fluoride (PVDF) membrane [30]. Immunoblot analysis was done with monoclonal anti-acetylated tubulin 6-11B-1 antibodies (T7451, Sigma-Aldrich), at a dilution of 1:100. Total tubulin was detected with polyclonal anti-tubulin α antibody (SAB4500087, Sigma-Aldrich) at a 1:500 dilution. Secondary anti-mouse and anti-rabbit HRP (ab97023, ab6721, Abcam, respectively) conjugated antibodies were used at a 1:1000 dilution. Densitometry was done using ImageJ (NIH), by measuring the intensity of all pixels comprising the band of interest [31]; the relative density of the bands from each experiment was recorded.

### 2.9. Cell-to-Cell Fusion

Ten thousand ED cells were divided into two batches: the first was nucleofected with luciferase-expressing plasmid pT7-EMC-Luc where luciferase is under control of the T7 promotor, while the second was nucleofected with T7-polymerase expressing plasmid pCAGT7 [32]. The nucleofection of ED cells was performed using cell line Nucleofector kit V (Lonza, Basel, Switzerland) [33], following the manufacturer’s instructions. After 24 h of incubation at 37 °C, cells that received the luciferase plasmid were infected with EHV-1 at a MOI of 1 for 3 h, and then treated for 3 h with one of the small GTPase inhibitors. The cells were washed with PBS, trypsinized, and mixed with the ED cells nucleofected with the T7 polymerase plasmid. Cells were incubated together for 24 h in the presence or absence of inhibitors. All cells were lysed, and the luciferase activity was quantified using the Luciferase Assay System (E1500, Promega, Madison, United States) and a microplate reader according to the manufacturer’s protocol.

### 2.10. Statistical Analysis

Cell viability, plaque assays and flow-cytometry-based studies were analyzed with GraphPad software. A one-way ANOVA analysis was done under the assumption of normal distribution. All values were compared to the control, and Duntett’s correction for multiple comparisons was used. Significance value was set to *p* = 0.05.

## 3. Results

### 3.1. Cdc42 and Rac1 Inhibitors Reduce EHV-1 and EHV-4 Infection

We have shown previously that the binding of EHV-1, but not EHV-4, to cell surface α4β1 integrins results in the release of cytosolic Ca^2+^, which is required for virus fusion with the plasma membrane [15]. The mechanism of how cytosolic Ca^2+^ can facilitate the fusion of the virus envelope with the plasma membrane is still unknown. Activation of small GTPases was described to be downstream of Ca^2+^ release [16,34], and might play a role in virus entry and subsequent transport within the cells. Therefore, we investigated the role of different small GTPase in virus infection.

**Figure 1:**
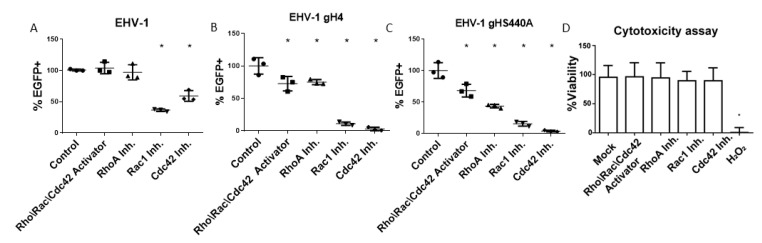
Cytotoxicity and inhibition of virus infection using GTPase inhibitors. Inhibition of equine herpesvirus EHV-1 (**A**), EHV-1 gH4 (**B**), or EHV-1 gHS440A; (**C**) infection in the presence or absence of different inhibitors determined by flow cytometry. Equine dermal (ED) cells were treated for three hours with small GTPase inhibitors or the activator as indicated and infected at a multiplicity of infection (MOI) of 1. eGFP expression was used to determine the percentage of infected cells relative to mock-treated cells. (**D**) Cytotoxicity assay (WST-1) in ED cells for mock- and hydrogen peroxide-treated cells (as a negative and positive control, respectively) as well as different GTPase inhibitors. Data are presented as means with standard deviation (S.D.) of three independent experiments, and normalized to the mean of mock-treated and infected cells. One-way ANOVA test followed by Dunnett’s multiple comparisons test; * *p* < 0.05.

ED cells were treated for 3 h with one of the small GTPase-specific inhibitors NSC 23766 (Rac1 inhibitor), ML-141 (Cdc42 inhibitor), purified C3 transferase (RhoA inhibitor) or the small GTPase Rho\Rac\Cdc42 activator. Cells were then infected in a synchronized manner in the presence or absence of inhibitors or the activator. At three hours post-infection (hpi), cells were analyzed by flow cytometry to determine the progress of infection. In a three-hour infection assay, we observed a significant reduction of EHV-1, which binds to α4β1 integrins, as well as EHV-1 gH4 or EHV-1 gHS440A (these two viruses cannot bind to α4β1-integrin) infections after treatment of cells with Rac1 and Cdc42 inhibitors. The infection of EHV-1 was significantly reduced in cells treated with Rac1 (~60%) and Cdc42 inhibitors (~40%) (Figure 1B). The inhibitory effect of Rac1 and Cdc42 inhibitors was more pronounced in the case of EHV-1 gH4 and EHV-1 gHS440A, where it resulted in an ~80% (Rac1 inhibitor) and ~95% (Cdc42 inhibitor) reduction of infection. The Rho\Rac\Cdc42 activator and RhoA inhibitors had no effect on EHV-1 infection; however, they significantly reduced the infection (30–60% reduction) of EHV-1 gH4 and EHV-1 gHS440A, the two mutants that cannot bind to integrins [19], (Figure 1C,D). EHV-4 was not suitable for such an assay, due to the slow onset of eGFP expression that needed long incubation times to be expressed at measurable levels. It is worth mentioning that neither the inhibitors nor the activator had any toxic effect on the cells (Figure 1A).

### 3.2. Small GTPases Facilitate Infection and Cell-to-Cell Spread

**Figure 2:**
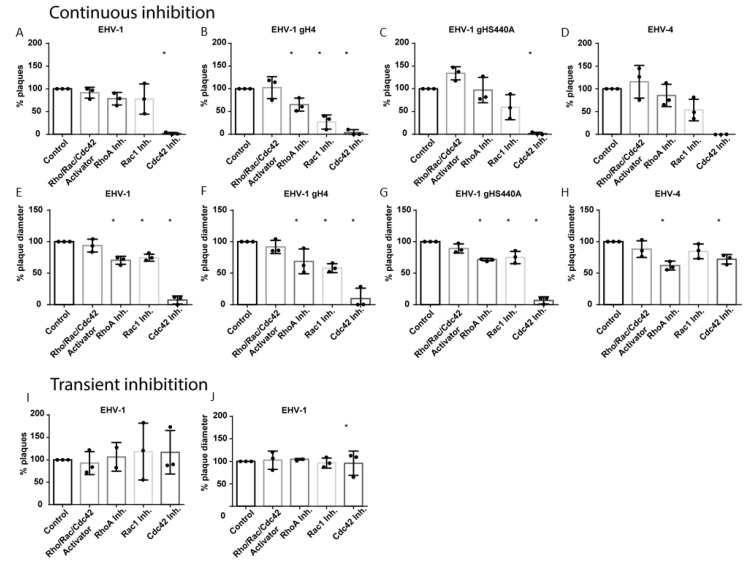
EHV-1 infection and cell-to-cell spread is influenced by small GTPases. Plaque reduction assay of EHV-1 (**A**,**E**,**I**,**J**), EHV-1 gH4 (**B**,**F**), EHV-1 gHS440A (**C**,**G**) and EHV-4 (**D**,**H**) in ED cells following inhibition of small GTPases. ED cells treated for three hours with inhibitors of small GTPases were infected with 100 PFU of the indicated virus for one hour, and then subjected to citrate treatment, overlaid with media that was inhibitor-free (**I**,**J**), or contained inhibitor (**A**–**H**) for 48 h. The number of plaques (**A**–**D**, **I**) was counted with an inverted fluorescent microscope and plaque diameters (**E**–**H**,**J**) were measured using ImageJ (NIH). Black circles “•” represent data obtained from each replicate. In case of plaque diameter panels (**F**–**H**), each circle represents average plaque diameter of 50 plaques (or all available) in a replicate; when there were no plaques (but only single infected cells) in a replicate, plaque diameter was considered to be zero. Data are presented as means with S.D. of three independent experiments and normalized to the mean of the control (virus infection without treatment). One-way ANOVA test followed by Dunnett’s multiple comparisons test; * *p* < 0.05.

ED cells were treated with inhibitors for three hours before infection. The virus was allowed to attach to cells for 1 h at 4 °C, then the temperature was shifted to 37 °C to facilitate virus entry for another hour. The cells were subjected to citrate treatment to eliminate all non-penetrated virus particles. Later, cells were overlaid with DMEM, containing 0.5% carboxymethyl cellulose in the presence or absence of inhibitors, until analysis at 48 hpi. Treatment of cells with ML-141 (Cdc42 inhibitor) resulted in a significant reduction of EHV-1 plaque formation (less than 3% of plaques; Figure 2A). On the other hand, Rac1 or RhoA inhibitors or the Rho\Rac\Cdc42 activator had no effect on plaque numbers (Figure 2A). Next, we measured the diameter of plaques produced in the presence of small GTPase inhibitors. The diameter was significantly reduced, and approximately 20% smaller in the presence of RhoA and Rac1 inhibitors, or approximately 90% smaller in the presence of the Cdc42 inhibitor (Figure 2E).

Similar to EHV-1, the number of plaques formed by EHV-1 gH4, EHV-1 gHS440A and EHV-4 was significantly diminished by the Cdc42 inhibitor (Figure 2B–D). RhoA and Rac1 inhibitors significantly reduced plaque numbers for EHV-1 gH4. While not statistically significant, a similar trend was observed for EHV-1 gHS440A and EHV-4 (Figure 2B–D). The diameter of plaques formed by EHV-1 gH4, EHV-1 gHS440A and EHV-4 was significantly smaller in the presence of RhoA and Cdc42 inhibitors (Figure 2F–H). It is noteworthy that, after using Cdc42 inhibitor, in particular, the number of plaques was significantly reduced; however, we were able to analyze the diameter of all available plaques in each replicate (see Materials and Methods). The Rac1 inhibitor resulted in a significant reduction of plaque diameters in EHV-1-derived mutants EHV-1 gH4, EHV-1 gHS440A, but not in EHV-4 (Figure 2F–H).

Absence of the inhibitors from the overlay media did not alter the picture of infection compared to mock-treated cells (all viruses produced plaques that were similar in size and numbers) (Figure 2I,J). This result indicates that the observed inhibition of virus infections requires the presence of inhibitors throughout the infection. We concluded from our data that small GTPases are actively involved in the entry, but also at later steps of the virus infection cycle.

### 3.3. EHV-1 Activates Small GTPases Rac1 and Cdc42

**Figure 3:**
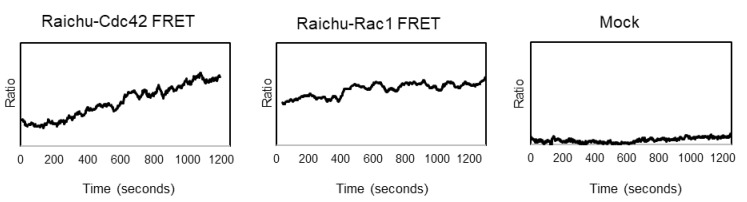
Small GTPase activation with ratiometric FRET in response to EHV-1 infection. Raichu-FRET biosensors were used to examine the activation status of small GTPases. ED cells were transfected with pRaichu-Rac1- or pRaichu-Cdc42-biosensor expressing plasmids. Twenty-four hours after transfection, EHV-1 RFP was added to ED cells and emissions of donor and acceptor was simultaneously measured and later analyzed. The ratio of FRET/CFP (acceptor/donor) was calculated for the selected region of interest (transfected cell) and plotted as a moving average of 20 frames.

To get a direct measurement of the activation status of the small GTPases Rac1 and Cdc42 during virus infection, a FRET-based system was employed. ED cells were transfected with either the pRaichu-Rac1- or pRaichu-Cdc42 FRET constructs. The expressed large protein is a single polypeptide chain that consists of four domains: the YFP domain and CFP domain at the termini, the Rac1 or Cdc42 GTPase domain, and the Ras-binding domain. The Ras-binding domain can only bind to small GTPase domain in its active form, i.e., when it is GTP bound. The interaction of the Ras-binding domain with the small GTPase domain results in conformational changes in the whole protein when the small GTPase is active. This interaction between the two central domains will then lead to conformational changes of the protein, and bring the CFP and YFP domains into close proximity and allow for the FRET effect to take place. We measured the activation of Rac1 and Cdc42 in ED cells in response to exposure with UV-inactivated EHV-1, using the FRET-based biosensors. ED cells expressing the FRET biosensors were imaged with 2 HZ frequency with two sensors, allowing for the simultaneous collection of the signals emitted by donor (CFP) and FRET (YFP), with an imaging duration of 20–25 min. At two minutes after the start of the imaging sequence, UV-inactivated EHV-1 was added to the cells. The mean intensity for the region of interest was measured, and the ratio of FRET/CFP was calculated and plotted as a moving average of 20 points. The measurement was repeated three times for each of the biosensors. We observed the activation of both (Rac1 and Cdc42) small GTPases in cells shortly after exposure to EHV-1 (Figure 3).

### 3.4. Tracking of Virus Transport in Cells

**Figure 4:**
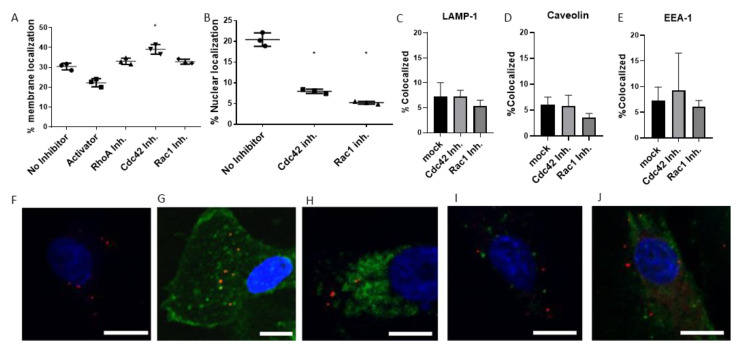
Effects of small GTPase inhibitors on virus transport. Inhibitor-treated ED cells were infected with EHV-1 at a MOI of 1, fixed at 30 min, 3 or 6 h after infection, and stained in one of the following ways. (**A**) ED cells fixed at 30 min post infection were stained with FITC-lectin, and the number of particles localizing with the plasma membrane was counted manually. (**B**) At 6 hpi, ED cells were fixed and stained with DAPI. (**C**–**E**) Colocalization of EHV-1 with endosomal markers in ED cells treated with inhibitors of the small GTPases Cdc42 and Rac1. Colocalization of EHV-1 with cellular compartments: cells were stained with only DAPI at 6 hpi. Exemplary images following staining: (**F**) DAPI stain at 6 hpi. (**G**) FITC-lectin 30 min after infection, (**H**) anti-LAMP-1 antibodies 3 hpi, (**I**) anti-EEA-1 antibodies 3 hpi, (**J**) or anti-caveolin antibodies 3 hpi. Scale bars represent 10 μm. DAPI is pseudocolored blue, EHV-1 particles as red, staining target (Lectin, LAMP-1, EEA-1, or Caveolin-1) as green. Data are presented as means with S.D. of three independent experiments and normalized to the mean (with S.D.) of the control (virus infection without inhibitor treatment). One-way ANOVA was employed, followed by Dunnett’s multiple comparisons correction; * *p* < 0.05.

The entry of EHV-1 into equine ED cells can either be through direct fusion with the plasma membrane, or after interaction with the α4β1 integrins that induce a signaling cascade, triggering the fusion process. Alternatively, if integrin binding is altered and the signaling cascade abrogated, EHV-1 is redirected to the endosomal pathway [15]. Virus particles are then transported inside the cell to the nucleus along microtubules [35], where replication takes place. We investigated whether small GTPases play a role in the virus transport that might lead to a reduction of infection, as seen in Figure 1. The transient effects of GTPase inhibitors on EHV-1 infection (Figure 2) indicated that GTPases play a role at steps of infection after cell entry. In a time-resolved fashion, we investigated three loci for the presence of virus particles after infection: (i) the plasma membrane at 30 min post infection, (ii) endosomes at three hours post infection, and (iii) nucleus or cytoplasm at six hours post infection. ED cells were treated with chemical inhibitors of small GTPases for three hours, then infected with EHV-1, and fixed after 30 min, 3 or 6 hpi. One-hundred virus particles were counted manually in a blinded fashion in three replicates for each setup. For the 30-min setup, each particle was counted as on cell borders (colocalized with the plasma membrane), as visualized by FITC-labeled lectin. The treatment of cells with Cdc42 inhibitors resulted in a small, yet significant, increase in the number of virus particles that were colocalized with the plasma membrane when compared to mock-treated cells (Figure 4A). A decrease in colocalization with the plasma membrane was determined after treatment of cells with Rho\Rac\Cdc42 Activator (Figure 4A). We did not observe a change in numbers of plasma membrane-localizing viruses after treatment with Rac1 or Rho inhibitors (Figure 4A). Additionally, we did not record any increase in virus colocalization with endosomes, lysosomes, or caveolae at 3 hpi in inhibitor-treated cells when compared to mock-treated cells (Figure 4C–E). We further investigated the efficiency of virion delivery to the nucleus in the inhibitor-treated ED cells, by counting the number of virus particles in the perinuclear zone and the cytoplasm. Both Cdc42 and Rac1 inhibitors resulted in a significant delay of virus transport to the nucleus when compared to virus transport in mock-treated cells: a significantly smaller number of virus particles reached the nucleus, and the majority remained in the cytoplasm at 6 hpi (Figure 4B). Taken together, these findings indicated that the small GTPases, Cdc42 and Rac1, play a role in the transport of the virus particles to the cell nucleus.

### 3.5. Rac1 and Cdc42 Activation Is Required for EHV-1-Induced Tubulin Acetylation

**Figure 5:**
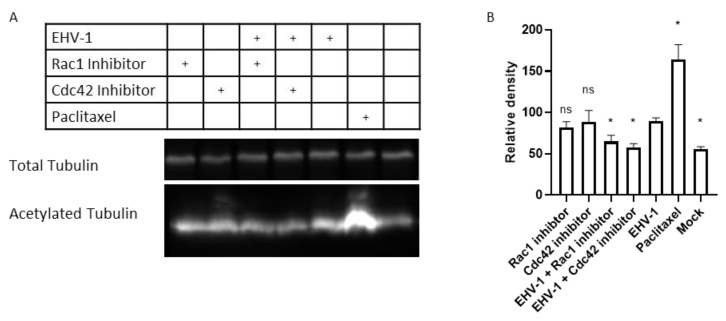
Tubulin acetylation by EHV-1 requires virus-activated GTPases. (**A**) Human 293T cells were treated with inhibitors or mock-treated for 3 h and infected for 60 min with EHV-1. Paclitaxel, a chemical inducing microtubule acetylation, was used to treat cells for one hour as a positive control. Cell lysates were used for immunoblotting with anti-total tubulin, and an antibody that is specific for acetylated tubulin. (**B**) Densitometry of the bands in (**A**) was done using ImageJ. The experiment was repeated three times. Data are presented as means with S.D. of three independent experiments and normalized to the mean (with S.D.) of the control (EHV-1 virus infection without inhibitor treatment); One-way ANOVA test followed by Dunnett’s multiple comparisons correction (each sample vs EHV-1); * *p* < 0.05.

Alphaherpesviruses utilize microtubule transport to reach the nucleus [36]. EHV-1, in particular, is transported by dynein motor proteins to the cell nucleus [28]. The acetylation of lysine 40 (K40) is an important posttranslational modification of α-tubulin that stabilizes microtubule structure [37,38,39] and increases dynein binding and motility [40]. EHV-1, as well as HSV-1, were described to induce microtubule acetylation, as the inhibition of acetylation resulted in reduced virus infectivity of both viruses [28,41]. To investigate whether small GTPases play a role in microtubule acetylation, we treated cells with small GTPases inhibitors prior to infection and determined tubulin acetylation levels with monoclonal antibodies by immunoblot analysis. We confirmed that EHV-1 induces microtubule acetylation at 1 hpi in 293T cells, through a mechanism that might involve the activation of Rac1 and Cdc42. We further found that both Rac1 and Cdc42 inhibitors significantly prevented the acetylation of the α-tubulin when compared to EHV-1-infected cells (Figure 5).

### 3.6. EHV-1-Induced Cell-to-Cell Fusion Is Dependent on Rac1 and Cdc42 Activation

**Figure 6:**
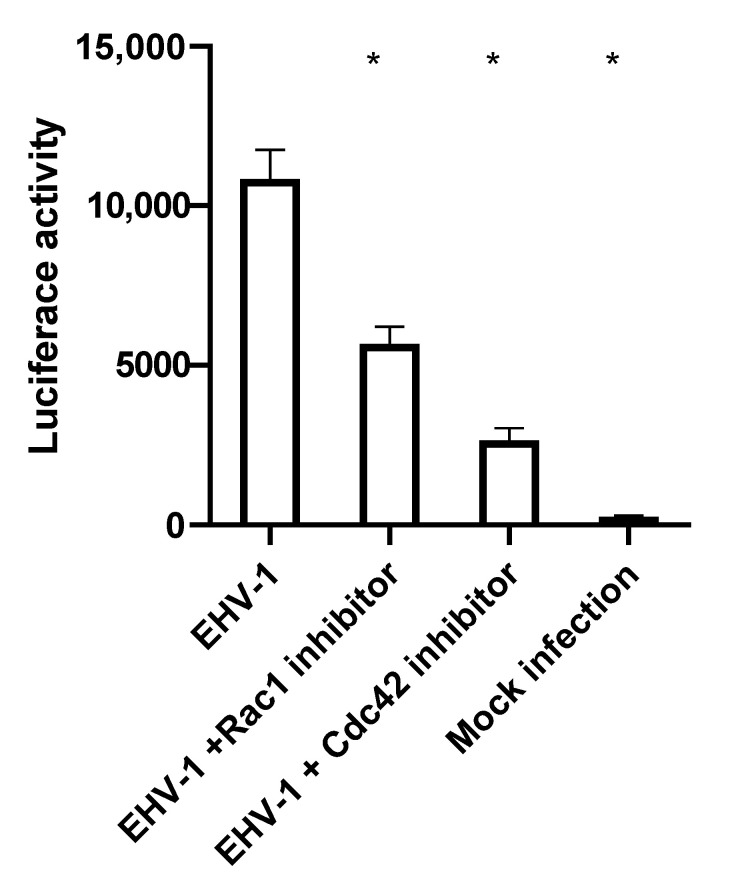
Small GTPases influence cell-to-cell fusion after infection. ED cells were nucleofected with pT7-EMC-Luc or T7 polymerase-expressing plasmid pCAGT7. pT7-EMC-Luc-nucleofected cells were infected with EHV-1 for three hours and then treated with inhibitors, or mock treated, for three hours. Both pT7-EMC-Luc-nucleofected infected-cells and pCAGT7-nucleofected non-infected cells were mixed and incubated for 24 h. Cells were lysed, and the levels of luciferase protein produced were measured as intensity of chemiluminescence. Data are presented as means with S.D. of three independent experiments (one-way ANOVA under assumption of normal distribution). All values were compared to the control (EHV-1 infection without inhibitor treatment), and Duntett’s correction for multiple comparisons was used; * *p* < 0.0001.

EHV-1 has the ability to trigger the fusion of infected with non-infected cells. The viral glycoproteins gB, gD and gH/gL that are expressed in infected cells mediate cell-to-cell fusion [42]. Small GTPases play a role in membrane remodeling, as well as the cytoskeletal rearrangements in infected cells [43,44] that govern the cell motility and transport efficacy inside the cell. To test whether small GTPases play a role in cell-to-cell fusion, luciferase-based cell-to-cell fusion assays were performed. To investigate the role of small GTPases on cell-to-cell fusion in an experimental infection set-up with chemical inhibitors, it was necessary to establish similar levels of infections first, since we already know that small GTPase inhibitors significantly reduce EHV-1 infection in ED cells. Thus, comparison of cell-to-cell fusion in a group of cells that have different infection levels would produce biased results. With the purpose of having inhibitor-treated cells with similar infection rates, we first infected ED cells with EHV-1 for three hours, before the inhibitors were added for another three hours. Levels of infection, as measured by eGFP expression at 6 hpi, were determined and showed no influence of the inhibitors on infection rates; these conditions were chosen for the cell-to-cell fusion assay. In this assay, two groups of ED cells were independently nucleofected with (i) a plasmid vector encoding luciferase under the control of T7 promotor, and (ii) a plasmid vector encoding T7 polymerase. In this experiment, the expression of luciferase will only occur in cells that also express T7 polymerase protein. In other words, the levels of luciferase expressed are functions of the number of cells that undergo cell-to-cell fusion. At 24 h after nucleofection, ED cells nucleofected with the luciferase carrying plasmid were infected with EHV-1 at a MOI of 1 for 3 h, and subsequently subjected to inhibitor treatment for another 3 h. Afterwards, the infected luciferase carrying cells and T7 polymerase-expressing uninfected cells were gently detached, mixed, and incubated for 24 h, in the presence or absence of inhibitors. At 24 h after mixing, cells were lysed, and the level of luciferase produced was measured via the chemiluminescence intensity of the lysate for each treatment. We found that the treatment of cells with either Rac1 or Cdc42 inhibitors led to a significant decrease in cell-to-cell fusion (Figure 6).

## 4. Discussion

Cellular processes are tightly regulated through intracellular signaling events. Small GTPases are signaling molecules that are known to play a role in large variety of cellular processes, including cytoskeleton remodeling, cell cycle regulation, motility, and other regulatory functions [3,6]. Furthermore, it has been shown that small GTPases are implicated in facilitating the entry and infection of several viruses [10,11,12]. Binding of EHV-1 gH to α4β1 integrins can induce signaling cascades inside the cell that lead to virus fusion with the plasma membrane [15]. In the current study, we have investigated the role of small GTPases during EHV-1 entry and throughout the infection cycle. Our data show that the small GTPases Cdc42 and Rac1 facilitate virus entry and promote efficient virus replication by (i) facilitating virion transport inside the cells through the regulation of α-tubulin acetylation and (ii) regulating the direct fusion of infected with uninfected cells, thereby enhancing the cell-to-cell spread. EHV-1 was able to induce Rac1 and Cdc42 activation, as demonstrated by a FRET analysis.

Rac1 and Cdc42 inhibitors had a strong inhibitory effect on EHV-1 infection. To investigate at which step of the replication cycle the virus was affected, we monitored virus trafficking inside the cell, starting from entry, microtubule transport to the nucleus, and ending with direct virus spread from the infected to uninfected cells. The inhibition of Cdc42 led to a moderate, but significant, increase in virus particles at the plasma membrane, indicating a delay in cellular entry. The inhibition of Rac1, on the other hand, had no effect on virus entry. In a previous study, Hoppe and coworkers reported that there was no effect on the cell entry of HSV-1 in MDCKII cells with inhibited Rac1 or Cdc42 GTPases [12]. On the other hand, the activation of Rac1 and Cdc42 was shown to be essential for the entry of dengue virus type-2 (DENV-2), since it relies on the formation of filopodia [45] and for the entry of pseudorabies virus via micropinocytosis in HeLa cells [46]. In contrast, one study of HSV-1 in keratinocytes showed that Rac1 and Cdc42 signaling pathways were not important [47]. However, Rac1 and Cdc42 are generally considered to be required for HSV-1 infection [12,48]. Both filopodia formation and virus fusion at the plasma membrane rely on actin remodeling, which in turn is regulated via small GTPases [49,50].

We also investigated if EHV-1 was redirected towards the endosomal pathway [51]. In the absence of Rac1 or Cdc42 signaling, there were no significant changes in virion localization in the endosomes, suggesting that Rac1 and Cdc42 play no role in the choice of entry pathway. Investigation of virion localization to the nucleus revealed that a smaller proportion of virions was present in the nucleus of cells treated with either Cdc42 or Rac1 inhibitors. These findings are consistent with the infection assay results (Figure 1) that also showed that the inhibition of Rac1 or Cdc42 led to a decrease in overall production of infectious EHV-1. Presumably, virus particles that cannot reach the nucleus are degraded in the cytoplasm by the proteasome, or are re-directed to lysosomes [36,52]. Alternatively, they may be sequestered in the cytoplasm in a quiescent state [53]. It was described for the vesicular stomatitis virus (VSV) that under conditions with reduced Cdc42 activation, the trafficking of viral particles from the ER to the Golgi was blocked, and the majority of VSV particles were trapped in the ER [54]. In the case of Kaposi’s sarcoma-associated herpesvirus (KSHV), the virus is able to activate cellular signaling and the acetylation of microtubules. The disruption of the small GTPase signaling cascade and the consequent absence of microtubule acetylation led to the reduction of virion transport, as measured by the presence of viral DNA in the nucleus [55]. We, therefore, surmise that the main reason for reduced infection rates by blocking Cdc42 or Rac1 is reduced virion trafficking in the host cell, caused by the inability of EHV-1 to induce α-tubulin acetylation.

The observation that cells treated with Cdc42 or Rac1 inhibitors were able to internalize similar numbers of viruses compared to mock-treated cells and produce similar numbers of plaques in inhibitor-free media (Figure 2) suggests that the activation of small GTPases is not essential for cell entry. However, it was clear that virions require the activation of these GTPases to efficiently reach the nucleus for replication [55]. It seems likely that GTPases affect virion transport in the cytoplasm, most likely by interfering with proper microtubule function.

Alphaherpesviruses are known to be transported along microtubules to the nucleus [36,56]. We, therefore, investigated the acetylation of microtubules during infection, and the possible role of GTPases in this step. The acetylation of microtubules is considered an indication of their stability and kinesin and dynein motility [37,38,40,57]. We showed that the inhibition of Cdc42 and Rac1 reduced the virus-induced acetylation of α-tubulin. Similar effects were reported for EHV-1, where the activation of ROCK1 is required for the acetylation of α-tubulin [28]. In the case of influenza A virus (IAV) infection, the activation of the RhoA small GTPase was reported to induce tubulin acetylation, which is presumably required for trafficking of IAV particle components [58]. Similarly, KSHV was able to induce RhoA activation, which leads to the acetylation of tubulin in endothelial cells [55]. The HSV-1 VP22 protein was previously reported to be responsible for tubulin acetylation and microtubule bundling, even in the context of transient transfection [59]. An impaired ability of virus particles to induce α-tubulin acetylation in the presence of GTPase inhibitors might explain the accumulation of virus particles in the cytoplasm, due to the reduced transport in infected cells under such conditions.

Herpesviruses induce cell-to-cell fusion to facilitate virus spread [60,61]. We found that the small GTPases Cdc42 and Rac1 play a role in the post-replicative stages of EHV-1 infection by facilitating cell-to-cell spread. Inhibition of both GTPases, reduced virus cell-to-cell spread and the ability of virus-infected cells to induce cell-to-cell fusion. This reduction of the fusogenic activity of infected cells could also be a direct consequence of the decrease in acetylation of tubulin, which has been shown to play a role in cell motility and cell-to-cell fusion [62,63], or reduce the formation of cytoplasm extensions that serve for viral transport to neighboring cells, or the ability of the microtubules to transport infectious particles across the cytoplasmic extension [64]. Additionally, small GTPase inhibitors can affect cell-to-cell fusion through other mechanisms, that were not investigated in this study, such as (i) the modification of intracellular trafficking may result in reduced gB and gH-gL (virus fusion machinery) presence in the plasma membrane, and thus reduced cell to cell spread [6,42]; (ii) changes in the rates of polymerization of actin filaments can lead to reduced cell-to-cell contact [65,66]; (iii) or even change aspects of the cell physiology independent of viral infection [48].

The infection of EHV-4 and the other recombinant EHV-1 that are unable to bind to integrins EHV-1 gH4 and EHV-1 gHS440A) was also affected in a fashion similar to that recorded for EHV-1, which we showed binds to integrins. These data suggested that the activation of the small GTPases play a role in a the signaling cascade independent of that induced by integrins [51]. Further studies will be needed to investigate the exact mechanistic role of small GTPases in EHV-1 and EHV-4 infection. Of specific interest is our observation that while the plaque number of EHV-4 was strongly reduced, the reduction of plaque size was less pronounced.

In conclusion, we have shown that EHV-1 activates small GTPases to facilitate the stabilization of α-tubulin and promote virion transport inside the cell and to neighboring cells via cell-to-cell fusion. Chemical inhibitors of the small GTPases Rac1 and Cdc42 precluded the virus-induced activation of small GTPases and ultimately resulted in impaired virus trafficking to the nucleus of infected cell. Furthermore, the reduced acetylation of the microtubules is likely the main reason of the observed phenomena. A deeper understanding of the mechanisms of the virus-host interactions of EHV-1 and its relatives in the *Alphaherpesvirinae* may provide the basis for the rational development of therapeutics or preventive measures against this important group of human and animal pathogens.
